# Editorial: Design, Synthesis, and Application of Novel π-Conjugated Materials—Part Ⅱ

**DOI:** 10.3389/fchem.2021.771438

**Published:** 2021-09-23

**Authors:** Zhicheng Dai, Taotao Ai, Qixin Zhou, Haichang Zhang

**Affiliations:** ^1^ Key Laboratory of Rubber-Plastics of Ministry of Education/Shandong Province (QUST), School of Polymer Science &Engineering, Qingdao University of Science and Technology, Qingdao, China; ^2^ School of Material Science and Engineering, Shaanxi University of Technology (SNUT), Hanzhong, China; ^3^ National Center for Education and Research on Corrosion and Materials Performance, Department of Chemical and Biomolecular Engineering, The University of Akron, Akron, OH, United States

**Keywords:** π-conjugated materials, organic field effect transistors, sensors, organic solar cells, electronics

During last few years, new π-conjugated materials have received more and more attention in the community due to their potential wide range of applications such as organic field effect transistors (OFETs), solar cells, sensors and so on. Among them, the works with respect to the design and optimization of π-conjugated molecules have been extensively investigated. This research topic includes 10 articles of reviews and original research works, which describe a series of novel π-conjugated materials along with various applications. These articles provide an overview of different types of π-conjugated materials and of how they are designed and characterized, thereby providing an overview of progress and development direction in this field.

Over the last decade, heptazine-based π-conjugated materials, including polymeric graphitic carbon nitride (g-C_3_N_4_) and corresponding small molecules, have attracted extensive attention by virtue of intriguing optoelectronic and photocatalytic properties in the fields of organic optoelectronics and photocatalysis. From the perspective of organic electroluminescence (EL), Li and co-workers reported an interesting monomeric heptazine derivative (HAP-3DF) which exhibits enhanced EL via n-π* transition character and exciplex-based thermally activated delayed fluorescence (TADF), respectively (Li et al.). The same group subsequently provided an overview of monomeric and polymeric heptazine-based π-conjugated materials for light-emitting. In this review, the metal ion-containing, polymeric g-C_3_N_4_-based, monomeric heptazine-based light-emitting materials and devices are systematically summarized, which is not only beneficial for the future molecular design of high-performance luminescent materials, but also for the acceleration of practical applications of heptazine-based materials and devices (Li et al.).

Normally, conjugated materials exhibit strong emission in their solution phase, but showing weak or quenched luminescence in the solid phase due to the aggregation. Ma et al. synthesized the molecules that contain tetrastyrene and benzimidazole structures to obtain molecules (TPEBZMZ) with strong aggregation induced luminescence (AIE) effect. The fluorescent nanofiber membrane, prepared by electrospinning TPEBZMZ and polylactic acid (PLA) blend solution, showed excellent and reversible acid-induced discoloration. This work provides not only a novel AIE material, but also a simple strategy to design the stimulus responsive fluorescent film sensor (Ma et al.).

As an interesting electron donor-acceptor material, diketopyrrolopyrrole (DPP)-based donor-acceptor conjugated materials have a very bright prospect in the application of electronic devices, typically in OFET due to their high charge transfer mobility. Zhou et al. reviewed the DPP, iso-DPP and their derivatives-based materials in OFETs, and mentioned that the hole transfer mobility based on the DPP polymers is up to 26 cm^2^ V^−1^ s^−1^. To obtain high-performance DPP-based semiconductor materials, the research direction should focus on not only the modification of the chemical structures on planar materials backbone and large π-convergence system, but also the molecules packing such as strong π-π stacking and aggregation, short molecular distance etc. (Zhou et al.). Bao’s group reviewed hydrogen-bonded materials in OFETs. The authors reviewed a series of small molecules and polymers with hydrogen-bonding association in the application of OFETs, indicating that hydrogen-bonds could not only enable the molecular reassembly to obtain a more ordered crystalline structure and improved π - π stacking, but also reduce the distance of the neighboring molecules and thus increase the molecular packing density. These behaviors could significantly enhance the charge transfer mobility (Shi et al.). Lu et al. also reviewed the conjugated materials in OFETs. and other applications such as organic solar cells (OSCs), sensors, and coating. This article pointed out that there are still a big room for the scientists to explore the highly selective and sensitive sensors. Conjugated polymer coating with multi-functions is also promising and interesting. In addition, the development of novel materials in OSCs with a broad range of light absorption and high charge mobility is still the research trend in the field (Lu et al. ).

OSCs, as high-quality next-generation energy transfer devices, have attracted extensive attention by researchers. Among them, fullerene acceptors are being replaced by non-fullerene acceptors because of their weak absorption and limited structural modification. Lin et al. synthesized a non-fullerene electron-acceptor cyclized by selenium branched chain based on ring fusion perylene diimide (PDI) tripolymer, and obtained a device with V_OC_ up to 1.12 V. Although the maximum power conversion efficiency (PCE) in this work is only 1.6%, it still provides a feasible idea for seleniding other compounds to improve the performance of OSCs in the future (Lin et al.). Another type of potential photovoltaic device is perovskite solar cells (PSCs). Hole transfer layer (HTL) plays a crucial role in achieving high performance of PSCs. However, the most used HTLs require the dopant to enhance the PCEs of PSCs due to their inherent low conductivity, leading to the decrease of the stability of the PSCs. Thus, the development of dopant-free hole transfer materials (HTM) is one main research trend. Deng et al. reviewed a series of dopant-free HTMs and pointed out that the molecular design concept for high-performance dopant-free HTMs is enlarging the π-conjugation system, increasing the planarity of molecular backbone, and introducing the functional atoms/groups to achieve interfacial interaction between the HTM layer and the perovskite layer, and, which results in self-assembly within the HTMs (Deng et al.).

A suitable amount of fluoride anion is beneficial for our daily life, but excessive fluoride ion is harmful for human health and chemical engineering as well. Therefore, the detection of fluorine ions in organic media is an important research topic. Deng et al. designed n-tertbutyldimethylsyl substituted diodocarbazole (CA-TBMDS) as a colorimetric sensor for fluoride anion sensors in organic solution. The study showed that CA-TBMDS is a highly sensitive and selective for fluoride anion with a color change from colorless to yellow. The color change could be attributed to the principle of intermolecular proton transfer between the amide units and the fluoride ion. This work pointed out that N-TBMDS units containing organic dyes can be used to produce fluoride anion sensors (Deng et al.).

π-Conjugated polymers are usually prepared by polymerization. Thus, novel methods for the preparation of conjugated polymers have received considerable attention. Tang et al. reported a new strategy for the preparation of conjugated polymers by rearrangement. As their report shows, by using a simple one-step method, which treated polybutadiene with n-butyllithium and *N,N,N′,N′*-tetramethylethylenediamine, polymers with a large number of conjugated double bonds were prepared. This contribution opens a new pathway to the synthesis of π-conjugated polymers (Tang et al.).

In this Research Topic, five research articles and five review articles regarding π-conjugated materials have been collected, which hint that the molecular chemical structures indeed play a key role in the performance of their applications. In future, it is believed that more materials with π - combination will be used in a wide range of application areas as the key design idea. Therefore, the design and improvement of π-conjugated materials will be a very promising research direction and will make a significant impact on the development of clean energy and environmental protection.

